# Supplementary figures and images for: High expression of HSP90 is associated with poor prognosis in patients with colorectal cancer
Source: PeerJ. 2019 Oct 31;7:e7946. doi: 10.7717/peerj.7946 (PMC6825748; doi:10.7717/peerj.7946)

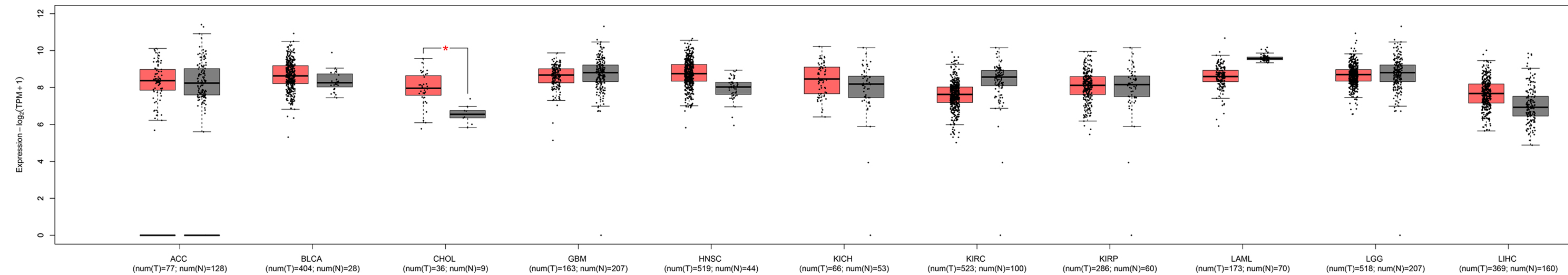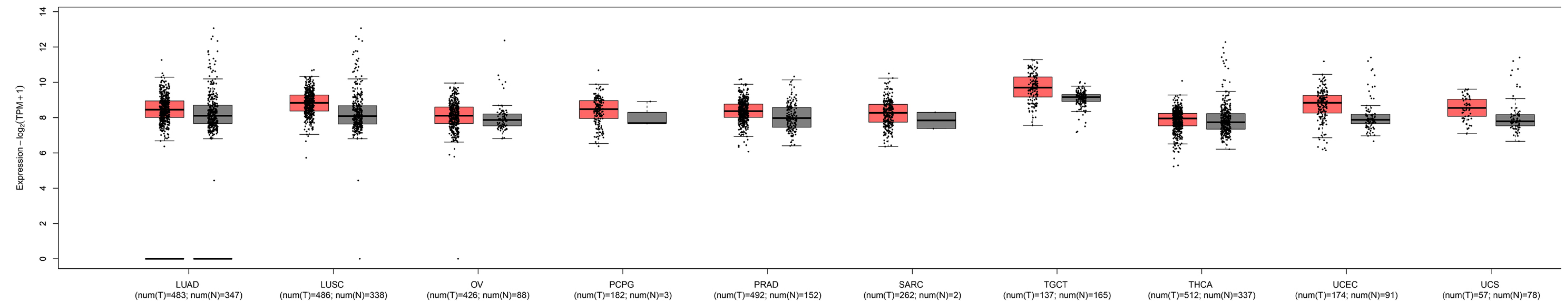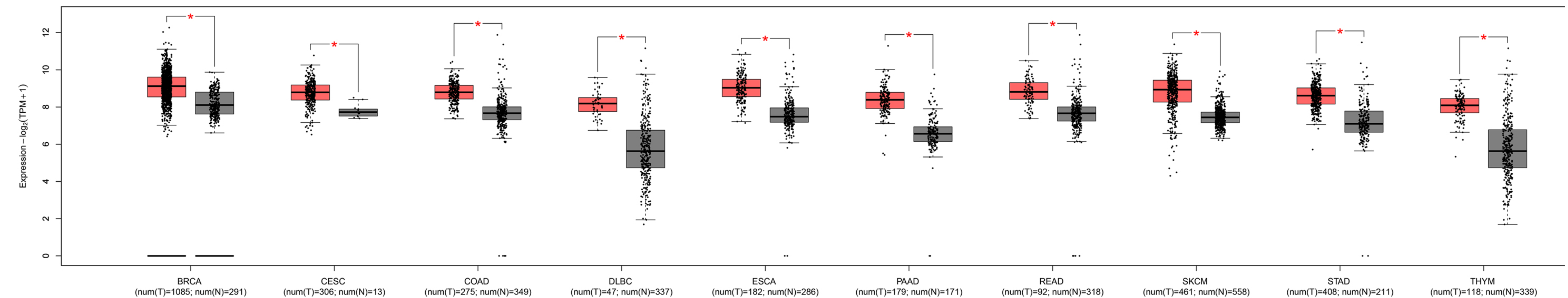

Supplement: Supplemental Information 1 [file peerj-07-7946-s001.pdf]

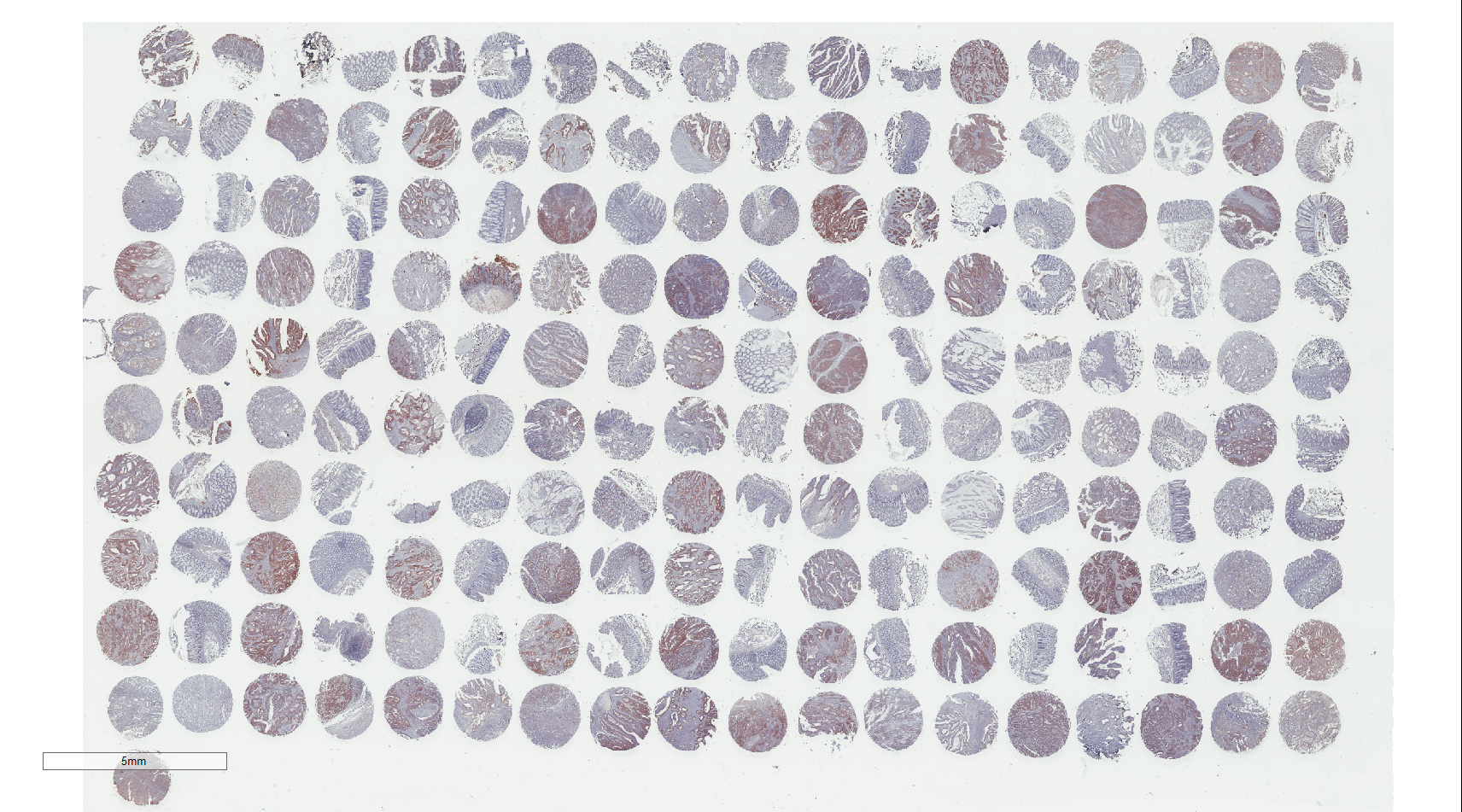

Supplement: Supplemental Information 6 [file peerj-07-7946-s006.png]
